# Translucent colonies and gram-negative diplococci: Meningococcal pneumonia masquerading as *Moraxella* pneumonia

**DOI:** 10.1016/j.idcr.2025.e02265

**Published:** 2025-05-12

**Authors:** Ryuichi Minoda Sada

**Affiliations:** a Department of Transformative Protection to Infectious Disease, Graduate School of Medicine, The University of Osaka, Osaka, Japan; b Department of Infection Control and Prevention, Graduate School of Medicine, The University of Osaka, Osaka, Japan; c Center for Infectious Disease Education and Research, The University of Osaka, Osaka, Japan; d Department of General Internal Medicine, Tenri Hospital, Tenri City, Nara, Japan

**Keywords:** *Neisseria meningitidis*, *Moraxella catarrhalis*, Pneumonia, Gram stain, Culture

## Abstract

• The patient presented with pneumonia and gram-negative diplococci on sputum smear.
• *Neisseria meningitidis* and *Moraxella catarrhalis* are both gram-negative diplococci.
• Both bacteria can cause pneumonia but can be differentiated by culture.
• Colonies of *N. meningitidis* are mucoid and translucent.
• Colonies of *M catarrhalis* are firm and white.

A 72-year-old man without relevant past medical history presented with a 5-day history of productive cough with white sputum, followed by fever and exertional dyspnea one day prior to admission. He lived alone and was independent in daily activities. There were no reported exposures to ill individuals, recent travel, or animal contacts. On examination, vital signs were: blood pressure 135/82 mmHg, heart rate 92 bpm, respiratory rate 30 bpm, temperature 37.7 °C, and oxygen saturation 92 % on room air. He was alert, with no neurological abnormalities, and notably, there were no signs of meningeal irritation such as neck stiffness or jolt accentuation. Auscultation revealed coarse crackles in the right lower lung field. Laboratory findings revealed leukocytosis (WBC 10,600/μL), elevated C-reactive protein (20.8 mg/dL), and hypoxemia (PaO2 59.8 mmHg). Chest radiography demonstrated right lower lobe consolidation with air bronchograms, consistent with pneumonia. Sputum Gram stain demonstrated numerous neutrophils and gram-negative diplococci (GNDC) (Fig. 1), initially suggesting *Moraxella catarrhalis*, a common cause of community-acquired respiratory infections. However, culture showed poor growth on blood agar and translucent, mucoid colonies on chocolate agar (Fig. 2), atypical for *M. catarrhalis*. Microbiological identification confirmed *Neisseria meningitidis* serogroup W-135, leading to a diagnosis of meningococcal pneumonia. The patient received intravenous ceftriaxone (2 g daily) for seven days, with full clinical and radiographic recovery. Blood cultures were negative, and lumbar puncture was not performed due to the absence of neurological symptoms or signs. Because of the transmissibility of *N. meningitidis* via respiratory droplets, droplet isolation precautions were implemented throughout hospitalization. His nine-year-old grandson, who had engaged in close contact, including kissing the patient shortly before admission, received rifampicin prophylaxis (10 mg/kg twice daily for two days). Fortunately, the grandson remained asymptomatic, and no secondary cases occurred among hospital staff or other contacts, indicating effective infection control measures.

**Fig. 1:**
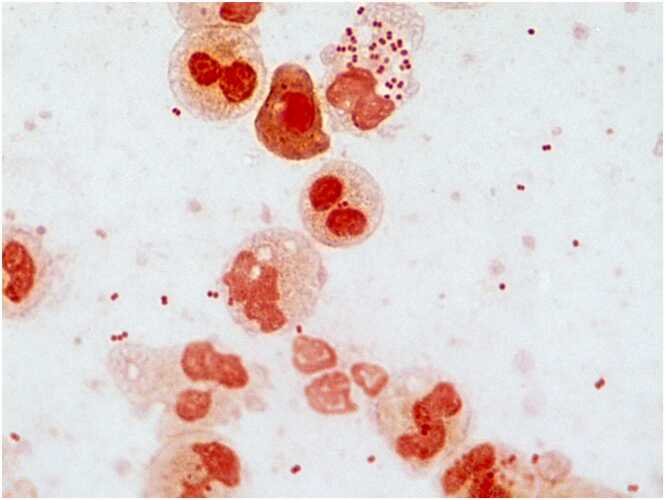
Gram stain of sputum on admission (× 1000) showing numerous polymorphonuclear leukocytes and gram-negative diplococci.

**Fig. 2:**
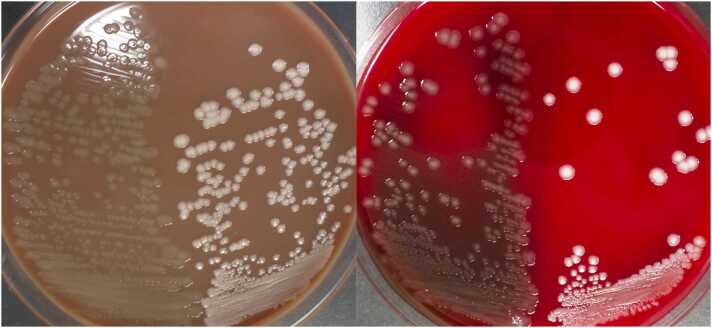
Sputum culture. Left: 5 % sheep blood agar; right: chocolate agar. The left side of each plate shows *Neisseria meningitidis* from this case; and the right side shows *Moraxella catarrhalis*. Note the mucoid, translucent appearance of *N. meningitidis* compared with the firm, white colonies of *M. catarrhalis*.

Meningococcal pneumonia is a rare manifestation of invasive meningococcal disease, accounting for approximately 0.26 % of bacterial pneumonias in Japan [1], [2]. Unlike meningitis or septicemia, isolated pneumonia may lack systemic symptoms such as headache or altered mental status. While GNDC in sputum typically suggests *M. catarrhalis*, clinicians should consider *N. meningitidis* especially when culture characteristics include poor growth on blood agar and translucent, sticky colonies on chocolate agar—findings that help clearly distinguish between the two. These findings highlight that early recognition and appropriate isolation are crucial to prevent transmission and ensure favorable outcomes [3].

## CRediT authorship contribution statement

**Ryuichi Minoda Sada:** Writing – review & editing, Writing – original draft, Visualization, Supervision, Conceptualization.

## Ethical approval

According to Japanese national guidelines, institutional review board approval is not required for single-patient case reports.

## Consent

The patient gave written informed consent for publication of his case details and related clinical images.

## Funding Source

No financial support was received from public institutions, private companies, or nonprofit organizations for the preparation of this case report.

## Conflict of Interest

None.

## Declaration of Competing Interest

The authors declare that they have no known competing financial interests or personal relationships that could have appeared to influence the work reported in this paper.
